# Efficacy and Safety of Multivisceral Resection for Ovarian Cancer: A Comparative Study of Gynecologic Oncologists and Multidisciplinary Surgeons

**DOI:** 10.1002/cam4.71671

**Published:** 2026-04-06

**Authors:** Luxin Ye, Qi Xue, Xianzhong Cheng, Ping Liu, Qian Zhao, Jiahui Chen, Xuening Wang, Yourong Chen, Xia Xu, Wenwen Guo, Jing Ni, Xiaoxiang Chen

**Affiliations:** ^1^ Department of Gynecologic Oncology The Affiliated Cancer Hospital of Nanjing Medical University, Jiangsu Cancer Hospital, Jiangsu Institute of Cancer Research, Jiangsu Key Laboratory of innovative Cancer Diagnosis & Therapeutics Nanjing Jiangsu China; ^2^ Gynecological Department Jiangsu Province Nanjing Gaochun People's Hospital Nanjing Jiangsu China; ^3^ Department of Chemotherapy Jiangsu Cancer Hospital, Jiangsu Institute of Cancer Research, The Affiliated Cancer Hospital of Nanjing Medical University Nanjing Jiangsu China; ^4^ Department of Pathology The Second Affiliated Hospital of Nanjing Medical University Nanjing Jiangsu China

**Keywords:** gynecologic oncologist, multidisciplinary team, multivisceral resection, ovarian cancer

## Abstract

**Background:**

Advanced ovarian cancer often presents with extensive pelvic and abdominal metastases, even malignant pleural effusion. Consequently, multivisceral resection has become the common surgical approach to achieve optimal resection of ovarian cancer. The present study aimed to evaluate postoperative complications and prognosis of multivisceral resection performed by the independent gynecologic oncologist team (GOT) or multidisciplinary team (MDT).

**Methods:**

The retrospective cohort study enrolled ovarian cancer patients who underwent surgery with multivisceral resection in Jiangsu Cancer Hospital. Patients were divided into two groups depending on the surgical team: the GOT and MDT groups. Patient baseline characteristics, surgical outcomes, postoperative complications, and long‐term prognosis were compared between the GOT and MDT groups.

**Results:**

Between January 2017 and June 2024, 299 ovarian cancer patients were included in this study: 83 in the GOT and 216 in the MDT group. The GOT group had shorter operating times (235 vs. 290 min; *p* < 0.001) and less blood loss (400 vs. 600 mL; *p* < 0.001) compared with the MDT group. There were no significant differences in postoperative complications and median progression‐free survival (PFS) (not reached (NR) vs. 22.8 months; HR = 0.818, 95% CI 0.507–1.320; *p* = 0.410) between the GOT and MDT groups. In multivariate analysis, HRD positive status was an indicator of a favorable prognosis (HR = 0.453; 95% CI 0.243–0.844; *p* = 0.013).

**Conclusion:**

The results suggest that ovarian cancer patients who underwent multivisceral resection performed by the independent gynecologic oncologist team are safe and feasible. Our team will continue to follow up to obtain more comprehensive survival data to validate this finding.

**Abbreviations**
ASA: American Society of Anesthesiologists physical status grade
BMI: body mass index
BRCA: breast cancer susceptibility gene
CCC: clear cell carcinoma
CDC: Clavien‐Dindo classification
CI: confidence interval
EC: endometrioid carcinoma
FIGO: International Federation of Gynecology and Obstetrics
GOT: gynecologic oncologists team
HGSC: high‐grade serous carcinoma
HR: hazard ratio
HRD: homologous recombination deficiency
ICU: intensive care unit
IDS: interval debulking surgery
IQR: interquartile range
MDT: multidisciplinary team
NR: not reached
PDS: primary debulking surgery
PFS: progression‐free survival
R0: no residual disease
R1: residual disease to less than 1 cm
R2: residual disease with a diameter > 1 cm
SD: standard deviation

## 1 Background

Ovarian cancer is one of the most lethal malignancies in women. According to the recent statistics, approximately 320,000 women were diagnosed with ovarian cancer and 210,000 died from the disease worldwide [1]. The majority of patients diagnosed with ovarian cancer were at an advanced stage and often developed extensive pelvic and abdominal metastases [2], even malignant pleural effusion [3]. The main treatments of ovarian cancer are cytoreductive surgery, platinum‐based chemotherapy and maintenance therapy [4], in which maximal cytoreductive surgery is the fundamental treatment. It was indicated that a 10% increase in maximal cytoreduction was associated with a 5.5% increase in median survival time [5]. The optimal goal of primary cytoreductive surgery is to reduce residual disease to less than 1 cm (R1). In particular, patients with no residual disease (R0) can benefit significantly [6]. To achieve complete cytoreduction, multivisceral resection is required, including bowel resection and/or appendectomy, stripping of the diaphragm or other peritoneal surfaces, splenectomy, partial cystectomy and/or ureteroneocystostomy, partial hepatectomy, partial gastrectomy, cholecystectomy, and/or distal pancreatectomy [7].

Multivisceral resection previously depended on the multidisciplinary team (MDT) due to its complexity [8]. However, with the continuous exploration of maximal cytoreduction, the gynecologic oncologists have become increasingly skilled in the ability to perform and manage postoperative complications. Compared to general surgeons, gynecologic oncologists have a better understanding of the biological behavior of ovarian cancer [9]. The available studies suggested that surgery performed by independent gynecologic oncologists is associated with better benefits [10]. In China, some gynecologic oncologists in professional cancer hospitals can independently perform multivisceral resection. However, most of them still need MDT to achieve satisfactory or R0 cytoreduction.

Nowadays, few studies have elucidated the effects of different surgical teams on multivisceral resections. The objective of our study was to evaluate the surgical outcomes, postoperative complications, and prognosis of multivisceral resection performed by independent gynecologic oncologists or the MDT group.

## 2 Methods

### 2.1 Study Population and Study Design

The retrospective study enrolled ovarian cancer patients who underwent multivisceral resection in primary treatment between January 2017 and June 2024 in Jiangsu Cancer Hospital. This study included patients who met the criteria for undergoing multivisceral resection and were pathologically diagnosed with ovarian malignancy postoperatively. Patients with poor general condition who underwent palliative surgery only were excluded. Multivisceral resection was defined as bowel resection, stripping of the diaphragm or other peritoneal surfaces, splenectomy, partial cystectomy and/or ureteroneocystostomy, partial hepatectomy, partial gastrectomy, cholecystectomy, and/or distal pancreatectomy [7]. All baseline and follow‐up information was obtained from the electronic medical record database. The study was approved by the ethics committee of Jiangsu Cancer Hospital.

The patient cohort was divided into two groups based on the surgical team responsible for performing multivisceral resection. The gynecologic oncologist team (GOT) group or MDT group was designated as the primary surgical team for multivisceral resection. The gynecologic oncologists in the GOT group were professionally trained and capable of performing highly complex ovarian cancer surgeries. General surgeons, urologists, or thoracic surgeons could be part of the MDT group. In the GOT group, the gynecologic oncology team undertook all the procedures of debulking surgery, including total abdominal hysterectomy with bilateral salpingo‐oophorectomy, upper abdominal surgery, and bowel resection. In the MDT group, the surgeons assisted the gynecologic oncologists in bowel surgery, upper abdominal surgery, and urological surgery.

### 2.2 Data Collection

The information of baseline characteristics included age at surgery, age at menarche, menopausal status, family history of cancer, body mass index (BMI, calculated as weight (kg)/[height (m)]^2^), timing of surgery (primary debulking surgery [PDS] or interval debulking surgery [IDS]), American Society of Anesthesiologists (ASA) physical status grade, clinical stage based on International Federation of Gynecology and Obstetrics (FIGO), histology, breast cancer susceptibility gene (BRCA) mutation status, homologous recombination deficiency (HRD) status, and the follow‐up. Patients were followed from the beginning of the postoperative period until July 2024, covering postoperative complications, subsequent treatment, and the presence of recurrence.

The data of surgical outcomes were also evaluated, incorporating the type of multivisceral resection, residual disease, timing of surgery, estimated blood loss, blood transfusion, postoperative anal exhaust days, postoperative intensive care unit (ICU) admission, postoperative ICU stays, hospital stays and administration of postoperative chemotherapy or maintenance therapy. Residual disease was classified as no residual disease (R0), residual disease to less than cm (R1), residual disease with a diameter > 1 cm (R2). The postoperative complications types were analyzed, including poor wound healing, pleural effusion, prolonged fever (> 38°C and > 3 days), subacute intestinal obstruction, abnormal sensation in the thighs, anastomotic leak, pancreatic leak, lower extremity venous thrombosis, postoperative infections, intestinal obstruction, vaginal leak and other complications. The complications of the surgery were graded according to the Clavien‐Dindo classification (CDC) [11].

### 2.3 Statistical Analysis

Student's *t*‐test or Wilcoxon rank sum test was employed for continuous variables, while chi‐squared or Fisher's exact test was utilized for categorical variables. The Cox proportional hazards regression model was applied to evaluate prognostic factors, and the hazard ratio (HR) with 95% confidence interval (CI) was calculated. All *P* values were two‐sided, and *p <* 0.05 were considered statistically significant. All analyses were performed with SPSS (Version 16.0) and GraphPad Prism (Version 8.0).

## 3 Results

### 3.1 Patient Characteristics

A total of 299 patients underwent multivisceral resection between January 2017 and June 2024. The GOT group performed 83 of 299 (27.8%), while the MDT group performed 216 of 299 (72.2%). In the GOT group, the mean age of the patients was 58.6 years. In the MDT group, the mean age of the patients was 58.0 years. The majority of patients were menopausal and diagnosed with FIGO stage III, a pathologic type of high‐grade serous carcinoma (HGSC). There was a significant difference between the two groups in the timing of surgery and ASA grade. The frequency of PDS in the GOT group was higher than in the MDT group (74.7% vs. 49.5%, *p* < 0.001). ASA grade III was more common in the GOT group (62.7%), while ASA grade II was more prevalent in the MDT group (55.6%) (*p* = 0.002). Among the known genetic testing information, most patients had a positive HRD status, although relatively few had BRCA mutations (Table 1).

**TABLE 1:** Patient characteristics.

Characteristic	GOT (*n* = 83)	MDT (*n* = 216)	*p*
Age at surgery (SD)	58.6 ± 9.0	58.0 ± 9.0	0.611
Age at menarche (SD)	15.2 ± 2.1	15.0 ± 1.7	0.409
Menopausal status, *n* (%)	68 (81.9)	174 (80.6)	0.787
Family history of cancer, *n* (%)	32 (38.6)	69 (31.9)	0.279
BMI (SD)	23.6 ± 3.1	23.5 ± 3.4	0.693
Timing of surgery, *n* (%)			< 0.001
PDS	62 (74.7)	107 (49.5)	
IDS	21 (25.3)	109 (50.5)	
ASA Grade, *n* (%)			0.002
II	29 (34.9)	120 (55.6)	
III	52 (62.7)	90 (41.7)	
IV	2 (2.4)	6 (2.8)	
FIGO Stage, *n* (%)			0.587
II	3 (3.6)	3 (1.4)	
III	57 (68.7)	141 (65.3)	
IV	21 (25.3)	55 (25.5)	
Unknown	2 (2.4)	17 (7.9)	
Histology, *n* (%)			0.458
HGSC	76 (91.6)	195 (90.3)	
LGSC	2 (2.4)	7 (3.2)	
EC	2 (2.4)	1 (0.5)	
CCC	1 (1.2)	2 (0.9)	
Other types	2 (2.4)	11 (5.1)	
BRCA status, *n* (%)			0.119
Mutation	13 (15.7)	26 (12.0)	
Wild	47 (56.6)	51 (23.6)	
Not tested	23 (27.7)	139 (64.4)	
HRD status, *n* (%)			0.401
Positive	38 (45.8)	54 (25.0)	
Negative	22 (26.5)	23 (10.6)	
Not tested	23 (27.7)	139 (64.4)	

Abbreviations: ASA, American Society of Anesthesiologists physical status grade; BMI, body mass index; BRCA, breast cancer susceptibility gene; CCC, clear cell carcinoma; EC, endometrioid carcinoma; FIGO, International Federation of Gynecology and Obstetrics; GOT, gynecologic oncologists team; HGSC, high‐grade serous carcinoma; HRD, homologous recombination deficiency; IDS, interval debulking surgery; LGSC, low‐grade serous carcinoma; MDT, multidisciplinary team; PDS, primary debulking surgery; SD, standard deviation.

### 3.2 Surgical Outcomes

In terms of the type of bowel resection, anterior resection or Dixon was the most common type of bowel resection in both groups. The MDT group showed a higher proportion of right hemicolectomy (12.5% vs. 1.2%; *p* = 0.001), Hartmann's procedures (9.7% vs. 2.4%; *p* = 0.034), and stoma formation (13.9% vs. 3.6%; *p* = 0.011) compared with the GOT group. Furthermore, the GOT group conducted more diaphragmatic lesion resections (27.7% vs. 13.0%; *p* = 0.002) and pelvic peritonectomy (7.2% vs. 0.9%; *p* = 0.007) than the MDT group (Table 2). Regarding surgical outcomes, the proportion of R0 after multivisceral resection showed no significant difference between the two groups (56.6% vs. 56.0%; *p* = 0.688). The GOT group experienced shorter operating times than the MDT group (235 min; range, 122–410 min vs. 290 min; range, 145–528 min; *p* < 0.001). In the assessment of blood loss, the GOT group exhibited a lower proportion of blood transfusion (44.6% vs. 57.9%; *p* = 0.039) and less blood loss (400 mL; range, 50–2100 mL in GOT group vs. 600 mL; range, 100–7000 mL in MDT group; *p* < 0.001) in comparison to the MDT group. Postoperative anal exhaust time was longer in the GOT group (5.0 days; IQR, 5.0–7.0 days in GOT group vs. 5.0 days; IQR, 4.5–6.5 days in MDT group; *p* = 0.001). A higher percentage of GOT group patients were admitted to ICU care compared with the MDT group (75.9% vs. 31.9%; *p* < 0.001). There was no significant difference between the two groups in hospital stays, selection of postoperative chemotherapy or maintenance therapy (Table 3).

**TABLE 2:** Type of multivisceral resection.

Characteristic	GOT (*n* = 83)	MDT (*n* = 216)	*p*
Anterior resection or Dixon, *n* (%)	48 (57.8)	105 (48.6)	0.153
Transverse colon resection, *n* (%)	2 (2.4)	7 (3.2)	1.000
Left hemicolectomies, *n* (%)	1 (1.2)	12 (5.6)	0.121
Right hemicolectomies, *n* (%)	1 (1.2)	27 (12.5)	0.001
Total colectomy, *n* (%)	1 (1.2)	1 (0.5)	0.479
Small bowel resection, *n* (%)	3 (3.6)	18 (8.3)	0.208
Hartmann's procedures, *n* (%)	2 (2.4)	21 (9.7)	0.034
Stoma formation, *n* (%)	3 (3.6)	30 (13.9)	0.011
Liver lesion resection, *n* (%)	11 (13.3)	19 (8.8)	0.251
Cholecystectomy, *n* (%)	0 (0.0)	9 (4.2)	0.067
Ureterectomy and/or Cystectomy, *n* (%)	1 (1.2)	3 (1.4)	1.000
Gastric lesions resection, *n* (%)	2 (2.4)	5 (2.3)	1.000
Splenectomy, *n* (%)	12 (14.5)	39 (18.1)	0.459
Diaphragm lesion resection, *n* (%)	23 (27.7)	28 (13.0)	0.002
Pelvic lymph node dissection, *n* (%)	10 (12.0)	37 (17.1)	0.280
Paraaortic lymph node dissection, *n* (%)	22 (26.5)	37 (17.1)	0.068
Lesser sac resection, *n* (%)	0 (0.0)	3 (1.4)	0.563
Pelvic peritonectomy, *n* (%)	6 (7.2)	2 (0.9)	0.007

Abbreviations: GOT, gynecologic oncologists team; MDT, multidisciplinary team.

**TABLE 3:** Outcomes of multivisceral resection.

Characteristic	GOT (*n* = 83)	MDT (*n* = 216)	*p*
Residual disease, *n* (%)			0.688
R0	47 (56.6)	121 (56.0)	
R1	33 (39.8)	77 (35.6)	
R2	3 (3.6)	18 (8.3)	
Operation time (range), min	235 (122–410)	290 (145–528)	< 0.001
Estimated blood loss (range), mL	400 (50–2100)	600 (100–7000)	< 0.001
Blood transfusion, *n* (%)	37 (44.6)	125 (57.9)	0.039
Postoperative anal exhaust time (IQR), day	5.0 (5.0–7.0)	5.0 (4.5–6.5)	0.001
Postoperative ICU admission, *n* (%)	63 (75.9)	69 (31.9)	< 0.001
Postoperative ICU stays (range), day	3.0 (1.0–6.0)	3.0 (1.0–6.0)	0.420
Postoperative hospital stays (range), day	10.0 (7.0–24.0)	11.0 (4.0–46.0)	0.905
Total hospital stays (range), day	15.0 (9.0–32.0)	14.0 (6.0–50.0)	0.133
Postoperative chemotherapy population, *n* (%)	82 (98.8)	209 (98.1)	1.000
Postoperative maintenance population, *n* (%)	41 (60.3)	109 (57.1)	0.644

Abbreviations: GOT, gynecologic oncologists team; ICU, intensive care unit; IQR, interquartile range; MDT, multidisciplinary team; R0, no residual disease; R1, residual disease to less than1 cm; R2, residual disease with a diameter > 1 cm.

This study included complications with CDC grade > I. No statistically significant difference was observed between the two groups in the occurrence of postoperative complications, complication CDC grades and complication types (Table 4). The most common postoperative complication was CDC grade II in the two groups (77.8% in the GOT group vs. 64.1% in the MDT group; *p* = 0.271). In the GOT group, the most common complications included intestinal obstruction (7.2%) and lower extremity venous thrombosis (6.0%). In the MDT group, the most common complications included poor wound healing (5.6%), intestinal obstruction (3.2%), and anastomotic leak (3.7%).

**TABLE 4:** Surgical complications.

Characteristic	GOT (*n* = 83)	MDT (*n* = 216)	*p*
Complications, *n* (%)	18 (21.7)	39 (18.1)	0.512
CDC Grade, *n* (%)			0.271
II	14 (77.8)	25 (64.1)	
III	2 (22.0)	12 (30.8)	
IV	0 (0.0)	2 (5.1)	
Poor wound healing, *n* (%)	1 (1.2)	12 (5.6)	0.121
Pleural effusion, *n* (%)	2 (2.4)	1 (0.5)	0.187
Prolonged fever (> 38°C and > 3 days), *n* (%)	1 (1.2)	2 (0.9)	1.000
Subacute intestinal obstruction, *n* (%)	3 (3.6)	6 (2.8)	0.712
Abnormal sensation in the thighs, *n* (%)	0 (0.0)	1 (0.5)	1.000
Anastomotic leak, *n* (%)	0 (0.0)	8 (3.7)	0.112
Pancreatic leak, *n* (%)	1 (1.2)	1 (0.5)	0.479
Lower extremity venous thrombosis, *n* (%)	5 (6.0)	5 (2.3)	0.147
Postoperative infections, *n* (%)	2 (2.4)	5 (2.3)	1.000
Intestinal obstruction, *n* (%)	6 (7.2)	7 (3.2)	0.201
Vaginal leak, *n* (%)	0 (0.0)	3 (1.4)	0.563
Others, *n* (%)	1 (1.2)	5 (2.3)	1.000

Abbreviations: CDC Grade, the grade of Clavien–Dindo classification; GOT, gynecologic oncologists team; MDT, multidisciplinary team.

### 3.3 Survival Outcomes

**FIGURE 1:**
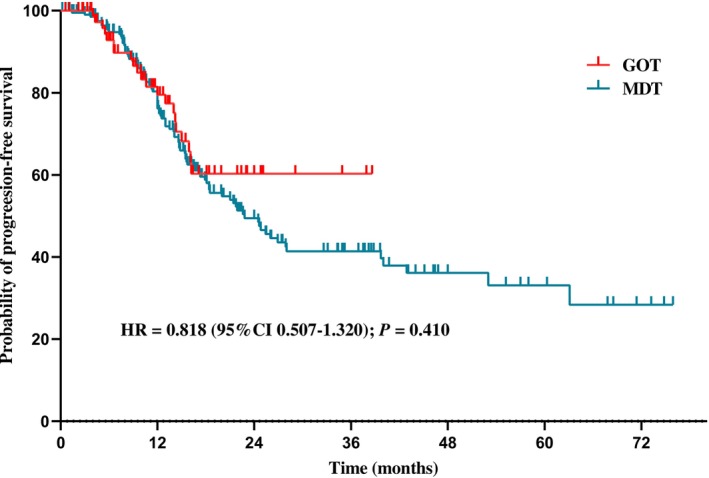
Kaplan–Meier Estimates of Progression‐Free Survival.

The median follow‐up time was 13.4 months. Between the GOT and MDT groups, no significant difference in median progression‐free survival (PFS) was observed (not reached (NR) vs. 22.8 months; HR = 0.818, 95% CI 0.507–1.320; *p* = 0.410) (Figure 1). In addition, ASA grade IV (HR = 18.627; 95% CI 3.965–87.502; *p* < 0.001), IDS (HR = 1.594; 95% CI 1.103–2.302; *p* = 0.013), and R1 resection (HR = 1.493; 95% CI 1.011–2.205; *p* = 0.044) were poor prognostic factors but were not significant in multivariate analysis. In multivariate analysis, HRD positive was considered an indicator of a favorable prognosis (HR = 0.453; 95% CI 0.243–0.844; *p* = 0.013) (Table 5).

**TABLE 5:** Univariate and multivariate analysis of prognostic factors for progression‐free survival.

	Univariate			Multivariate		
	HR	95% CI	*p*	HR	95% CI	*p*
Surgery by				—	—	—
MDT	1 (ref)					
GOT	0.818	(0.507, 1.320)	0.410	—	—	—
Age ≤ 60	1 (ref)					
Age > 60	1.361	(0.941, 1.968)	0.101	—	—	—
ASA Grade	—	—	0.001	—	—	—
II	1 (ref)					
III	1.092	(0.748, 1.594)	0.648	—	—	—
IV	18.627	(3.965, 87.502)	< 0.001	—	—	—
FIGO Stage	—	—	0.712	—	—	—
II	1 (ref)					
III	2.250	(0.311, 16.257)	0.422	—	—	—
IV	2.139	(0.288, 15.863)	0.457	—	—	—
Histologic type						
Non‐ HGSC	1 (ref)					
HGSC	1.101	(0.591, 2.051)	0.762	—	—	—
Timing of surgery						
PDS	1 (ref)					
IDS	1.594	(1.103, 2.302)	0.013	—	—	—
Residual disease	—	—	0.092	—	—	—
R0	1 (ref)					
R1	1.493	(1.011, 2.205)	0.044	—	—	—
R2	1.566	(0.820, 2.993)	0.174	—	—	—
BRCA status						
Wild	1 (ref)					
Mutation	0.758	(0.370, 1.551)	0.448	—	—	—
HRD status						
Negative	1 (ref)					
Positive	0.453	(0.243, 0.844)	0.013	0.453	(0.243, 0.844)	0.013

Abbreviations: ASA, American Society of Anesthesiologists physical status grade; BRCA, breast cancer susceptibility gene; FIGO, International Federation of Gynecology and Obstetrics; GOT, gynecologic oncologists team; HGSC, high‐grade serous carcinoma; HRD, homologous recombination deficiency; IDS, interval debulking surgery; MDT, multidisciplinary team; PDS, primary debulking surgery; R0, no residual disease; R1, residual disease to less than 1 cm; R2, residual disease with a diameter > 1 cm.

## 4 Discussion

Maximal cytoreductive surgery is the cornerstone of surgical treatment for ovarian cancer and also the key to a favorable prognosis for patients. Approximately 40%–80% of advanced ovarian cancer patients require bowel resection [12], and more than half of patients develop metastases to the upper abdomen [13]. Therefore, multivisceral resection is particularly important in ovarian cancer surgery to achieve maximal cytoreduction. Multivisceral resection often requires MDT collaboration [14]. A European survey indicated that less than 30% of bowel resections are performed independently by gynecologic oncologists, with even less data available for upper abdominal surgery [15]. However, few studies have assessed the effect of multivisceral resection for ovarian cancer among surgeons, including the rate of complete gross resection, postoperative complications, and long‐term prognosis, which may affect postoperative decision‐making and patient management. In recent years, with the expansion of the capabilities of the gynecologic oncologists, our Gynecologic Oncology Center team of well‐trained gynecologic oncologists has been able to perform independent surgeries, such as total pelvic and combined abdominal surgery. Previous studies show no significant difference in postoperative complications between gynecologic oncologists and surgeons performing cytoreductive bowel resection for ovarian cancer [16, 17, 18]. In this study, we obtained consistent results. There were no differences in postoperative complications and prognosis of multivisceral resection performed in the GOT group compared with the MDT group, and the incidence of postoperative complications was acceptable. This study showed that multivisceral resection for ovarian cancer performed by gynecologic oncologists could be safe and feasible.

Gynecologic oncologists should be responsible for the overall management of ovarian cancer [19]. The gynecologic oncologist was the first person responsible for seeing patients with ovarian cancer and fully captured the individual differences in the preoperative preparation. Thus, the gynecologic oncologist should be at the helm of surgery and take a leading role in determining the surgical approach and scope of surgery, while the intraoperative situation would be constructive for the postoperative treatment. Available studies suggest that R0 resection for PDS has a better prognosis than R0 resection for IDS [20]. This study showed that there was a higher proportion of PDS in the GOT group. This may be due to the well‐trained gynecologic oncologists being more inclined to perform multivisceral resection at the time of PDS. This further implied that multivisceral resection relied on gynecologic oncologists to individualize treatment based on patient characteristics.

Existing research suggested that gynecologic oncologists need to upgrade their skills in bowel surgery for ovarian cancer and be able to perform the procedure [21]. The finding was consistent with previous reports that anterior resection or Dixon was the most common type of bowel resection in both groups [12, 21, 22]. The MDT group presented with a higher percentage of right hemicolectomies, Hartmann's procedures, and stoma formation. This suggests that gynecologic oncologists need surgical assistance for more specialized bowel procedures. A higher percentage of stoma formation may be related to the experience of the surgeon [16]. The GOT group showed a higher rate of pelvic peritonectomy (7.2% vs. 0.9%, *p* = 0.007), possibly indicating that gynecologic oncologists have more experience with the biological behavior of ovarian cancer when it spreads. Previous studies have found that the prognosis for complete resection of some pelvic metastases via pelvic peritonectomy is not significantly different from the prognosis for pelvic bowel resection [22, 23].

Previous studies have indicated that intraoperative bleeding could be effectively reduced if the gynecologic oncologist was familiar with the anatomic characteristics of bleeding‐prone areas and had mastered effective hemostatic techniques [9, 24]. Our study proved this by demonstrating that the GOT group was more advantageous in reducing estimated blood loss in patients with ovarian cancer. We found that procedures performed by gynecologic oncologists saved the surgeon consultation time and required less operating time. Preoperative and intraoperative characteristics were determining factors for postoperative ICU admission [25]. In the GOT group, more patients were admitted to the ICU, which might be associated with a higher proportion of patients with preoperative ASA grade III and the decision‐making preferences of gynecologic oncologists. It was also an approach for gynecologic oncologists to perform multivisceral resections to ensure the safety of the patient.

Anastomotic leak was not observed in the GOT group. A large sample retrospective analysis showed the occurrence of anastomotic leak for bowel surgery for ovarian cancer to be 0.4%–5.2% [17], and a multicenter retrospective study found the rate of anastomotic leak for bowel surgery for ovarian cancer to be 0%–15.2% [26]. In comparison, the incidence of anastomotic leak in this study was acceptable. The proportion of subacute intestinal obstruction within 30 days after surgery was 3.6% and 2.8% in the GOT and MDT groups, respectively. The frequency of intestinal obstruction after 30 days after surgery was 7.2% and 3.2%, respectively. Bakkum‐Gamez et al. reported that the incidence of postoperative intestinal obstruction after surgery for ovarian cancer was about 30.3% [27], and Kim et al. found that the occurrence of postoperative intestinal obstruction after surgery for ovarian cancer by gynecologic oncologists and surgeons was 18.6% and 19.8%, respectively [18]. Compared with existing studies, the incidence of postoperative intestinal obstruction in this study was acceptable, demonstrating that intestinal resection could be safely performed by both the GOT group and MDT group.

In upper abdominal surgery, the frequency of postoperative pancreatic leak was 1.2% and 0.5% in the GOT group and MDT group, respectively, and the occurrence of postoperative pleural effusion was 2.4% and 0.5%, respectively. A recent study indicates that the incidence of postoperative pancreatic leak was 18.8% [28] versus 24% in an earlier study [29]. In the DRAGON study, the occurrence of pleural effusion after diaphragmatic surgery with intraoperative thoracostomy tube placement was 18.2% compared to 65.9% for those without intraoperative thoracostomy tube placement [30]. In comparison, the incidence of postoperative complications in upper abdominal surgery in our study is acceptable. It is important to note that gynecologic oncologists must be able to recognize and manage complications from upper abdominal surgery early on in order to safely perform the procedure [13].

It was found that only the HRD positive status had a reduced risk of recurrence compared to the negative status in this study. Certain studies have proven that ovarian cancers with HRD positive status exhibit unique biological behavioral characteristics, often exhibiting peritoneal lesions with less infiltrative architecture and lymph node metastasis, suggesting a greater likelihood of complete resection [31]. This further supports that the biological behavior of ovarian cancer has an impact on surgical treatment, which is a benefit to gynecologic oncologists.

The limitations of the study include a short follow‐up, a single‐center and retrospective analysis, and the median PFS in the GOT group was not achieved. Additionally, the OS of the two groups was not compared. This is a preliminary exploratory study. Our research team will continue to follow up in subsequent phases to obtain sufficient survival data to validate these findings.

## 5 Conclusion

This study confirmed that multivisceral resection for ovarian cancer performed by gynecologic oncologists is both safe and feasible. Future multi‐center, prospective studies are expected to further demonstrate these findings.

## Author Contributions

Luxin Ye: Data curation, Formal analysis, Investigation, Writing – original draft. Qi Xue: Data curation, Investigation, Formal analysis, Writing – review and editing. Xianzhong Cheng: Methodology, Formal analysis, Funding acquisition, Writing – original draft. Ping Liu: Conceptualization, Data curation, Investigation, Writing – original draft. Qian Zhao: Data curation, Methodology, Formal analysis. Jiahui Chen: Methodology, Investigation, Formal analysis. Xuening Wang: Data curation, Investigation. Yourong Chen: Data curation, Validation. Xia Xu: Validation, Supervision. Wenwen Guo: Supervision, Funding acquisition. Jing Ni: Conceptualization, Funding acquisition, Supervision, Writing – review and editing. Xiaoxiang Chen: Conceptualization, Funding acquisition, Project administration, Writing – review and editing.

## Funding

This work was supported by grants from the National Natural Science Foundation of China (82302974), Natural Science Foundation of Youth Fund Projects of Jiangsu Province (BK20210976), Jiangsu Provincial Scientific Research and Health Project (ZD2022005), Jiangsu Provincial Scientific research and Health Project for Women and Children (F202004), 789 Outstanding Talent Program of the Second Affiliated Hospital of Nanjing Medical University (789ZYRC202080122), Clinical Science and Technology Climbing Program‐”Spark”Basic Research Project, The Affiliated Cancer Hospital of Nanjing Medical University (ZJ202213), Research Project of Jiangsu Cancer Hospital (RCQY202402), Yishan Research Project of Jiangsu Cancer Hospital (YSPY202401) and Qunfeng Project of Jiangsu Cancer Hospital (DFXK202504).

## Ethics Statement

This study was approved by the institutional review board of Jiangsu Cancer Hospital (KY‐2025‐030) in accordance with the Declaration of Helsinki. As a retrospective study, the informed consent requirement was waived.

## Conflicts of Interest

The authors declare no conflicts of interest.

## Data Availability

The data that support the findings of this study are available from the corresponding author upon reasonable request.
